# Supplementary material for: Miniaturized on-chip spectrometer enabled by electrochromic modulation
Source: Light Sci Appl. 2024 Sep 29;13:278. doi: 10.1038/s41377-024-01638-4 (PMC11438984; doi:10.1038/s41377-024-01638-4)
Supplement: Supplementary file 1 — Supplementary information for Miniaturized on-chip spectrometer enabled by electrochromic modulation [file 41377_2024_1638_MOESM1_ESM.docx]

**Supplementary Information**

**Miniaturized on-chip spectrometer enabled by electrochromic modulation**

Menghan Tian,^1,2^ Baolei Liu,^1,2*^ Zelin Lu,^1^ Yao Wang,^1^ Ze Zheng,^1^ Jiaqi Song,^1^

Xiaolan Zhong,^1*^ and Fan Wang^1*^

^1^ School of Physics, Beihang University, Beijing, 100191, China

^2^ These authors contributed equally to this work.

*Correspondence: [liubaolei@buaa.edu.cn](mailto:liubaolei@buaa.edu.cn); [zhongxl@buaa.edu.cn](mailto:zhongxl@buaa.edu.cn); [fanwang@buaa.edu.cn](mailto:fanwang@buaa.edu.cn)

**Table of contents:**

S1. The principles of electrochromic materials.

S2. The calculation of charge in electrochromic devices.

S3. The electrochromic performance of ECD.

S4. Transmission spectra in CIE color space diagrams.

S5. Example reconstructions under different detection modes.

S6. Example reconstructions under four detection modes

S7. Schematic of the optical setup.

S8. Photo of the miniaturized on-chip spectrometer.

S9. The reconstructed spectra of different spectra with a larger dynamic intensity range.

1. **The principles of electrochromic materials**

Tungsten oxide (WO_3_) is a cathodic electrochromic material with high optical transmission contrast and excellent electrochromic stability. As a cathodic electrochromic material, the WO_3_ film changes from transparent bleached state to blue colored state under the action of dual insertion of cations and electrons. It reversibly returns to the transparent state when applying reverse voltage and the ions and electrons are extracted. The basic chemical reaction equation of the electrochromic process can be expressed as ^[1]^:

$WO_{3}|_{bleached}+xM^{+}+xe^{-} \underset{\leftrightarrow}{} M_{x}\mathrm{WO}_{3}|_{colored}$ (1)

where $M^{+}$ represents the electrolyte ion, $e^{-}$ represents the electron, and *x* represents the number of moles of the embedded ion. The coloring mechanism of WO_3_ has long been highly controversial, and the most accepted theory is that WO_3_ and the embedded ions can generate $M_{x}\mathrm{WO}_{3}$ with a tungsten-bronze structure through reversible reaction. In contrast, the spectral absorption during the electrochromic process arises from the charge transfer or polariton transition between W ions of different valence states.

Nickel oxide is an anode electrochromic material, the ion and electrons embedding process is the bleaching process, showing a colorless transparent state; and the ion extracting process is the coloring process, which usually shows a brown or brown-black state. Nickel oxide films may involve multiple crystalline phases in the electrochromic process. Standard nickel monoxide (NiO) has a salt-rock lattice, with a lattice constant of 0.4178 nm and a single-crystal NiO density of 6.67 g cm^-3^. However, it is more difficult to obtain pure NiO, and the oxygen content is usually high. This oxygen leads to vacancies in Ni^2+^, which is why nickel oxide materials generally exhibit p-type semiconductor characteristics. Nickel oxide films can also have good electrochromic effects in non-aqueous electrolytes, such as LiClO_4_-PC solution, and the following reactions are generally accepted at present ^[2-4]^:

${Li}_{\alpha_{0}}{Ni}_{1-x_{1}}O\left( colored \right)+\alpha_{1}{Li}^{+}+\alpha_{1}e^{-}\leftrightarrow{Li}_{{(\alpha}_{0}+\alpha_{1})}{Ni}_{1-x_{2}}O(bleached)$ (2)

${Li}_{{(\alpha}_{0}+\alpha_{1})}{Ni}_{1-x_{2}}O(bleached)\leftrightarrow{Li}_{\alpha_{2}}{Ni}_{1-x_{3}}O\left( colored \right)+\alpha_{3}{Li}^{+}+\alpha_{3}e^{-}$ (3)

where *α_0_+α_1_=α_2_+α_3_*, the transition between Ni^2+^/Ni^3+^ alters the absorption of visible light, which is macroscopically manifested as a change of color or transmittance.

1. **The calculation of charge in electrochromic devices**

The corresponding charges can be obtained from the CV curves, and the corresponding formulae are as follows:

$Q=\int_{0}^{t} idt=\frac{1}{2v}\int_{0}^{t} idU=\frac{S}{2v}$ (4)

where *Q* is the amount of charge, *ΔU* is the voltage window, *S* is the area enclosed by the CV curve, and *v* is the voltage sweep rate.

1. **The electrochromic performance of ECD**


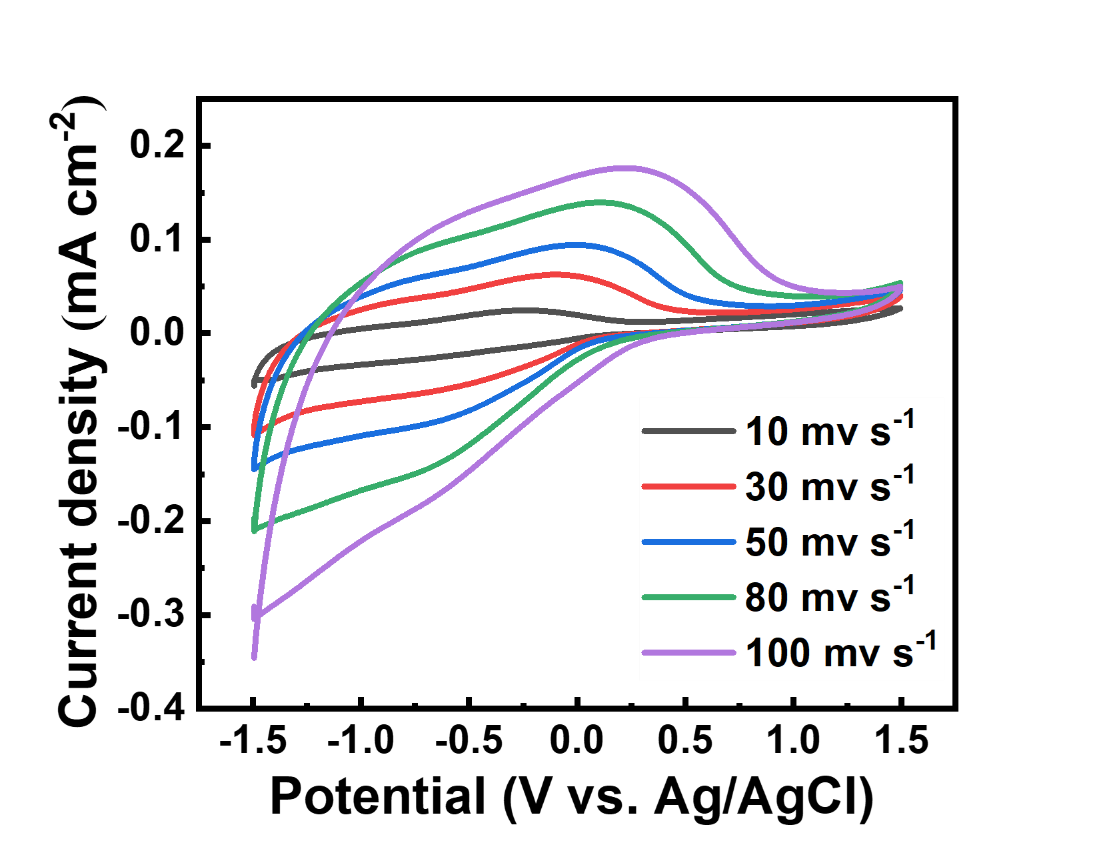
Fig. S1. The CV curves of ECD at 10, 30, 50, 80, and 100 mV s^-1^ scanning speeds, respectively.


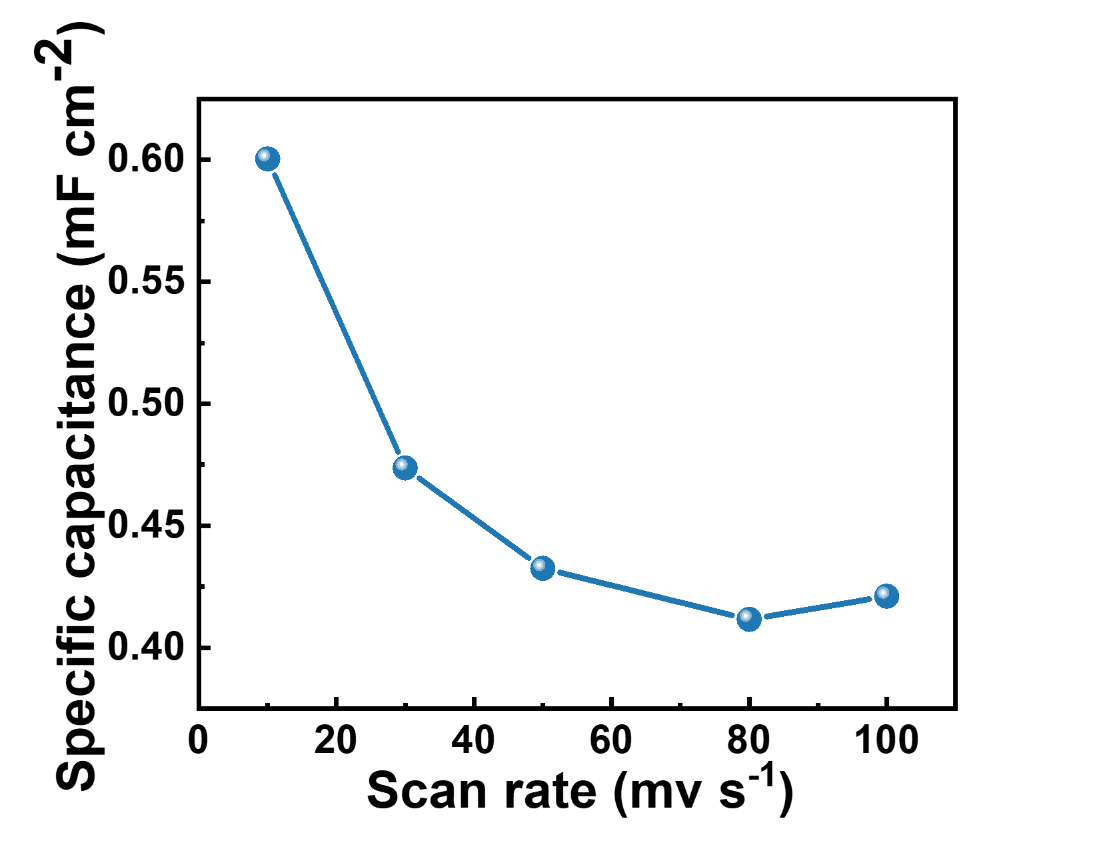
Fig. S2. The relationship between area energy density and sweep velocity.


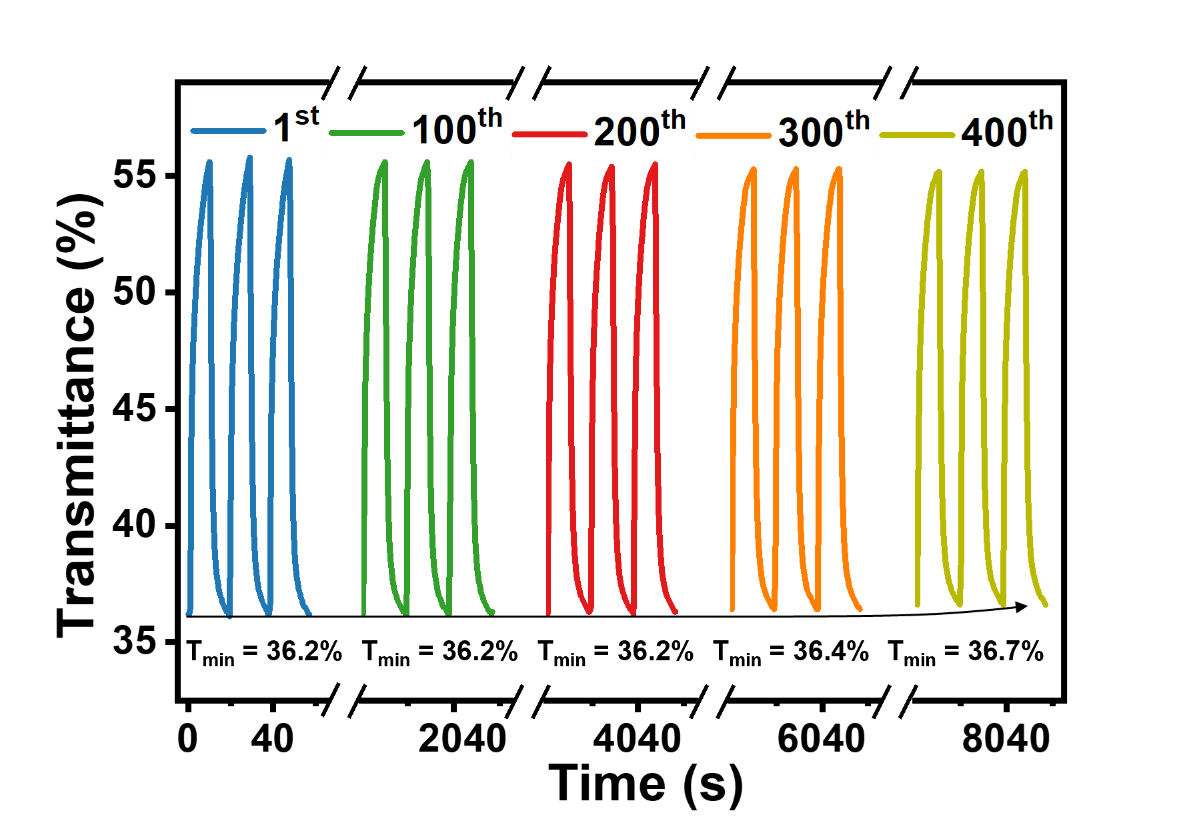
Fig. S3. The transmittance evolution curves of ECD, by applying a square wave voltage range in -1.5 V and 1.8 V.

1. **
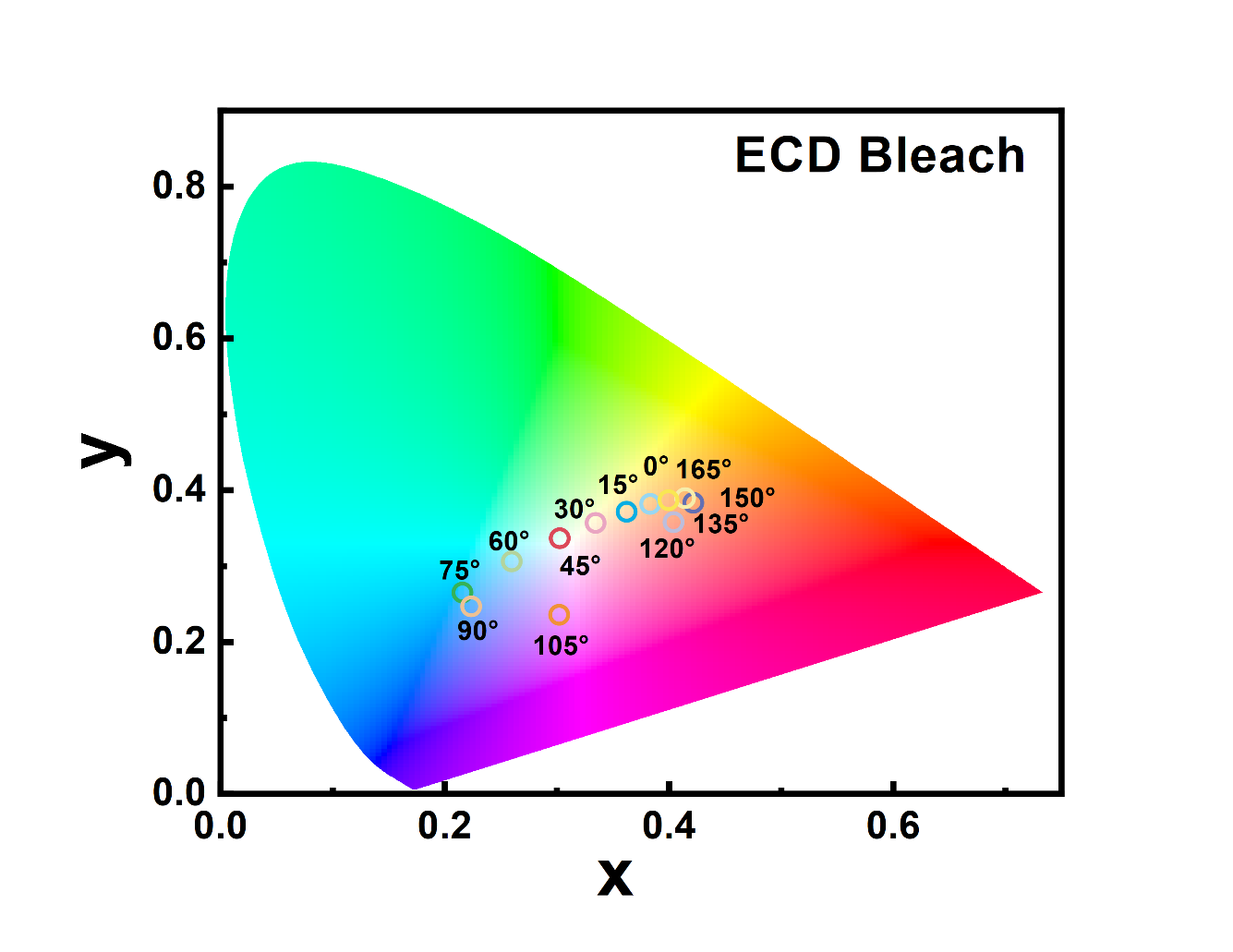
Transmission spectra in CIE color space diagrams**

Fig. S4. Transmittance spectra of ECD (bleached state) at different polarization angles in corresponding CIE color space diagrams.

1. **Example reconstructions under different detection modes**

**
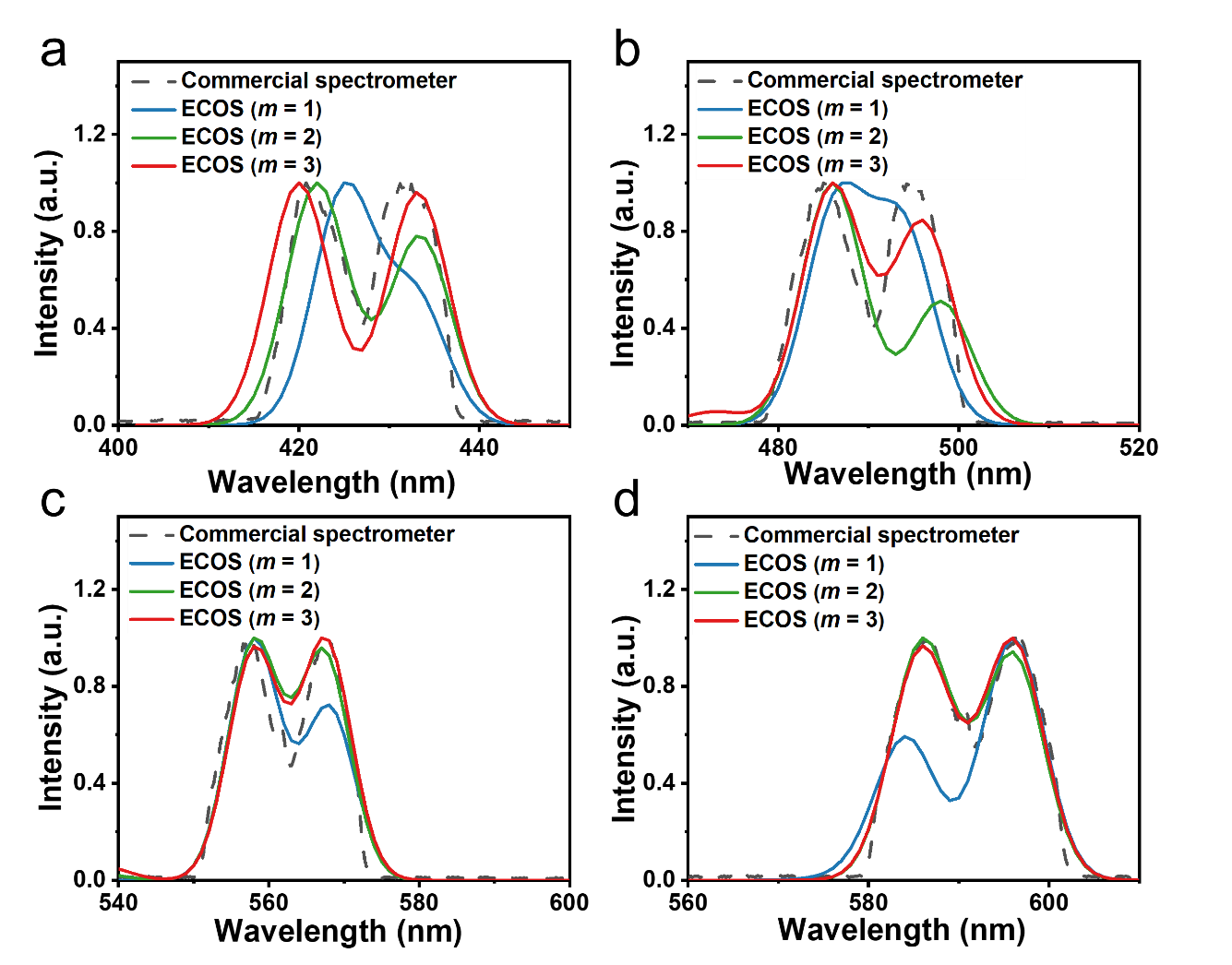
**Fig. S5. Reconstructions of the spectrum with two mixed peaks under different detection modes.

1. **Example reconstructions under four detection modes**


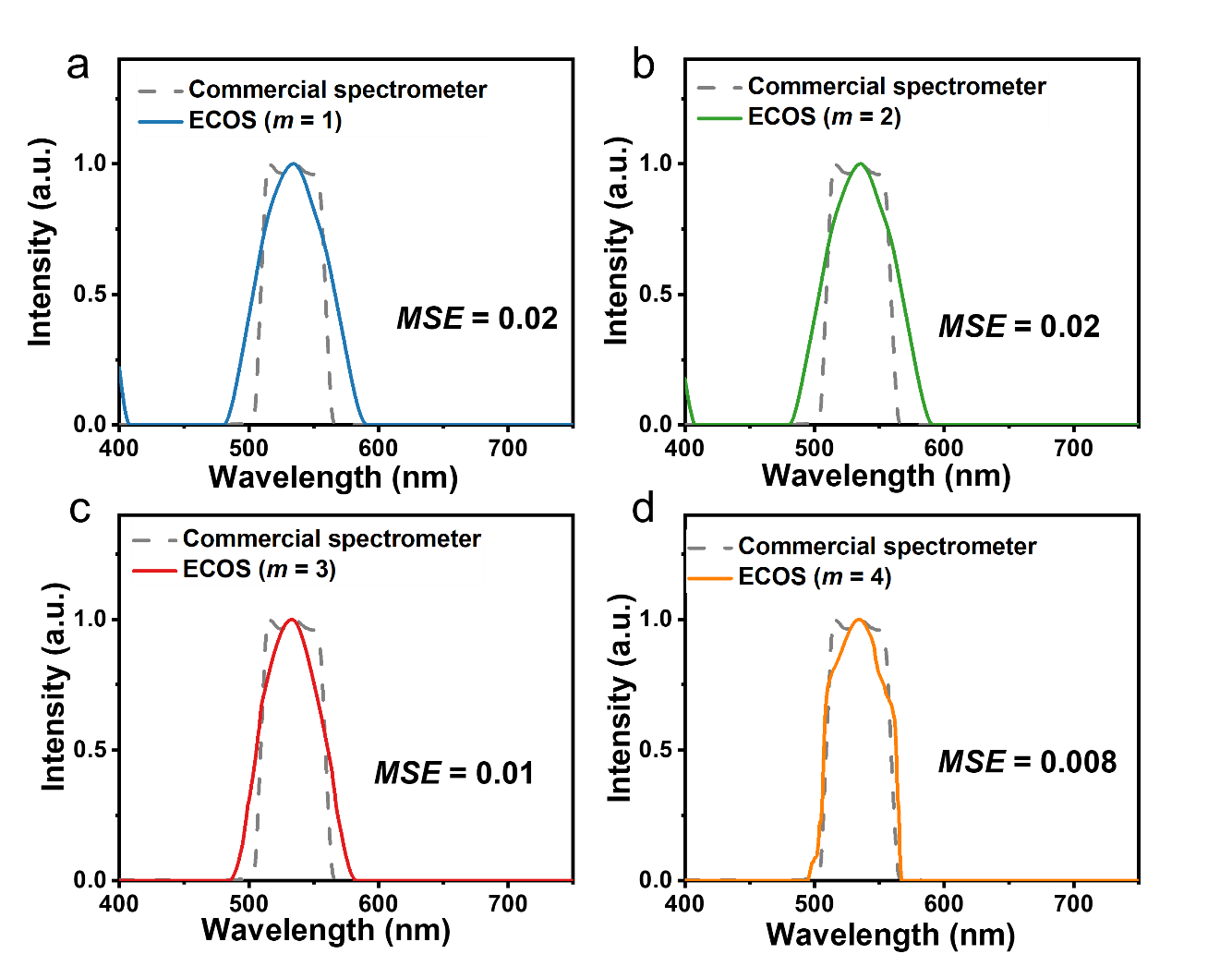
Fig. S6. Reconstructions of the spectrum with a 532 nm filter under four detection modes. Here *m* denotes different detection modes: ‘*m* = 1 (blue)’ refers to the usage of only ‘0 V’, ‘*m* = 2 (green)’ refers to the usage of ‘0 V’ and ‘-1.2 V’, ‘*m* = 3 (red)’ refers to the usage of ‘0 V’, ‘-1.2 V’, and ‘-1.5 V’, ‘*m* = 4 (orange)’ refers to the usage of ‘0 V’, ‘-1.2 V’, ‘-1.5 V’ and ‘-1.8 V’.

1. **Schematic of the optical setup**


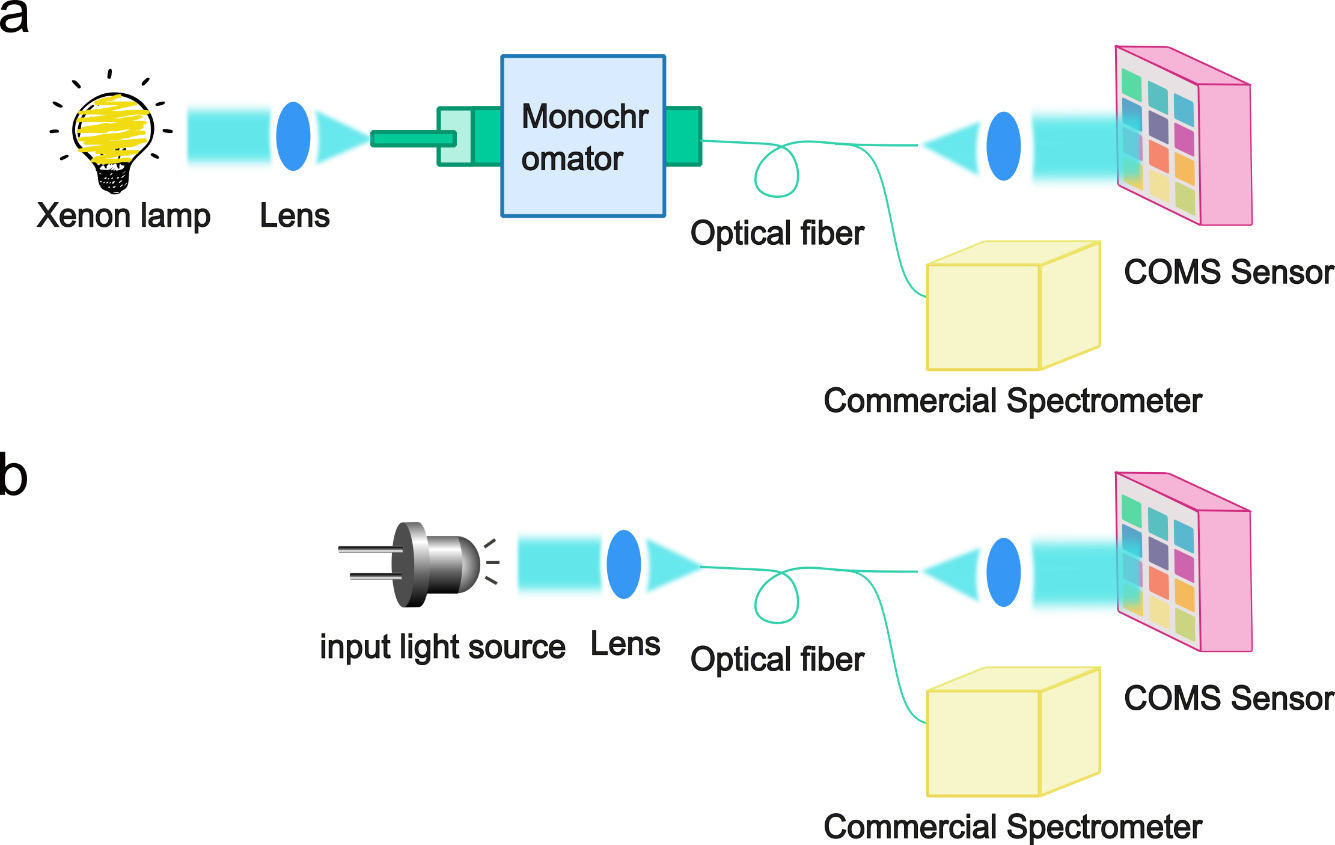


Figure S7. Schematic of the optical setup for calibration and measurement of the ECOS.

1.
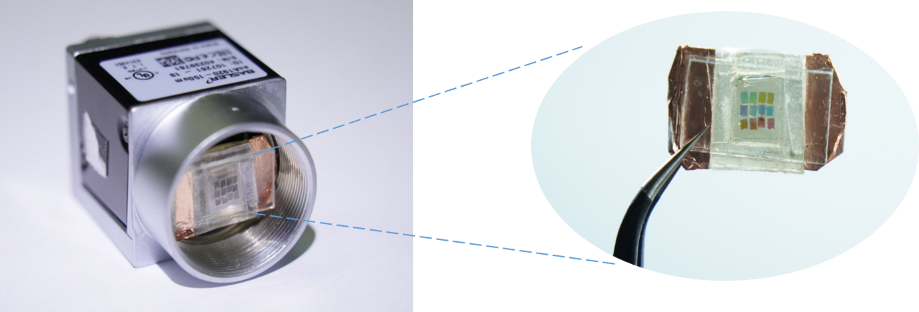
 **Photo of the miniaturized on-chip spectrometer**

Figure S8. Photo of the electrochromic computational on-chip spectrometer (ECOS).

1. **The reconstructed spectra of different spectra with a larger dynamic intensity range**

To explore the ability of electrochromic spectrometers to measure the spectrum with a larger dynamic intensity range, we reconstructed different spectra that have two peaks with the peak intensity ratios of 10:1 (Fig. S9a, c & e) or 100:1 (Fig. S9b, d & f) through numerical simulation. We use the transmission spectra of the ECD with different polarization angles at different voltages (0 V, -1.2 V, and -1.5 V) as the response functions (Fig.3) to reconstruct the input reference spectra. The reference spectra, which are presented as dotted lines in Fig. S9, have two peaks with wavelength differences of 100 nm or 200 nm. It can be seen that both the two peaks of the reference spectra with peak intensity ratios of 10:1 (Fig. S9a, c & e) have been reconstructed with good agreement. For the reference spectra with peak intensity ratios of 100:1 (Fig. S9b, d & f), both reconstructed peaks’ wavelength position matches the input spectra. The reconstructions for the peaks with higher intensities agree with the input, while the peaks with low intensities are not reconstructed well. The average MSE values for the peaks with lower intensities in the case of peak intensity ratios of 10:1 (Fig. S9a, c & e) or peak intensity ratios of 100:1 (Fig. S9b, d & f) are 0.004 or 0.031, respectively. It is noted that the wavelength values of these weak peaks can still be localized. It can be further optimized by modifying the spectral filters to improve the uncorrelation coefficients of the filter’s response spectra or modifying the reconstruction algorithms such as deep learning algorithms.


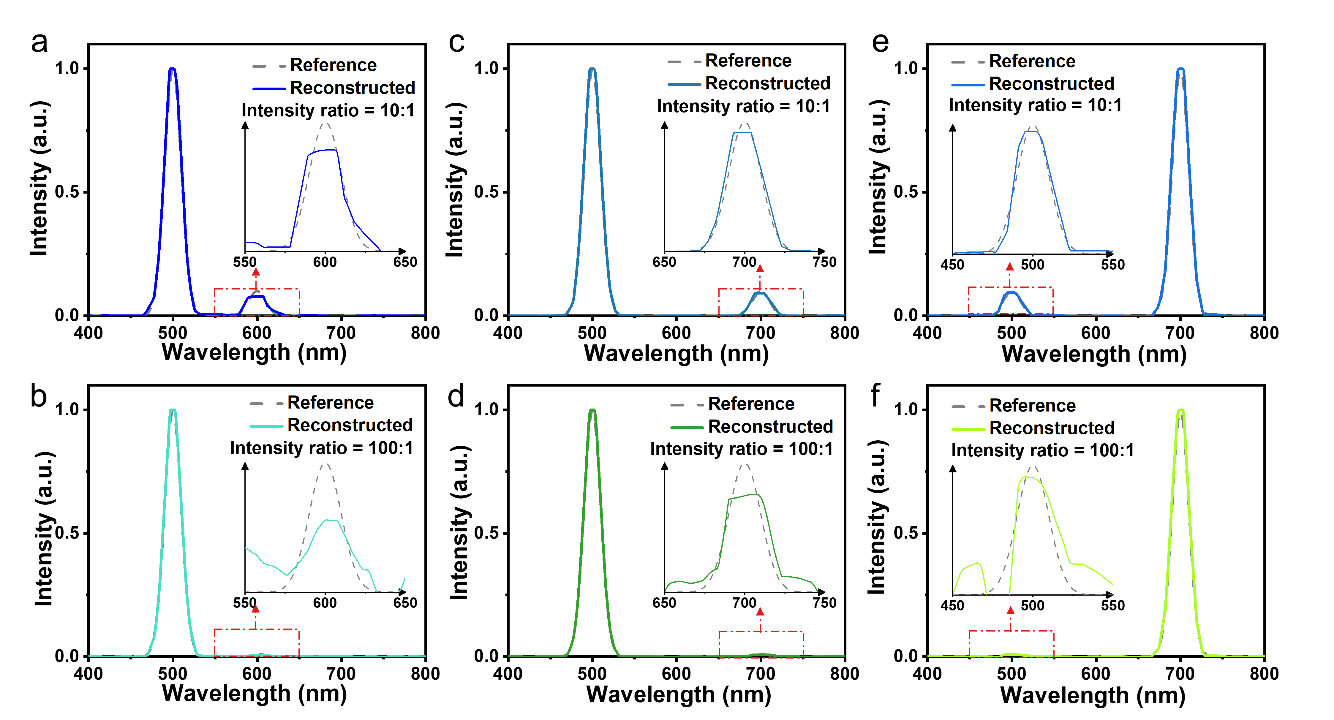


Figure S9. The numerical reconstructed spectra of different reference spectra have two wavelength peaks with the intensity ratio of 10:1(a, c, e) or 100:1(b, d, f) under detection modes (*m* = 3). The reference spectra are plotted in dotted lines.

**Reference:**

1. Granqvist C G. Electrochromic tungsten oxide films: Review of progress 1993–1998. Solar Energy Materials and Solar Cells, 2000, 60(3): 201-262.
2. Passerini S. The Intercalation of Lithium in Nickel Oxide and Its Electrochromic Properties. Journal of The Electrochemical Society, 1990, 137(10): 3297.
3. Campet G, Morel B, Bourrel M, et al., Electrochemistry of nickel oxide films in aqueous and Li^+^ containing non-aqueous solutions: an application for a new lithium-based nickel oxide electrode exhibiting electrochromism by a reversible Li^+^ ion insertion mechanism[J]. Materials Science and Engineering: B, 1991, 8(4): 303–308.
4. Lin Y-S, Chen P-W, Lin D-J, et al., Electrochromic performance of reactive plasma-sputtered NiO_x_ thin films on flexible PET/ITO substrates for flexible electrochromic devices[J]. Surface and Coatings Technology, 2010, 205: S216–S221.
